# Angina Pectoris

**Published:** 1909-12-11

**Authors:** W. J. Fenton

**Affiliations:** Assistant Physician, Charing Cross and Brompton Hospitals


### Hospital Clinics.

### ANGINA PECTORIS.

By W. J. FENTON, M.D. (Camb.), F.R.C.P. (Lond.); Assistant Physician, Charing Cross and Brompton Hospitals.

The case to which I wish to draw your attention to-day is that of a man about 55 years of age; he complains of paroxysms of acute pain beginning over the lower end of the sternum and radiating to the left shoulder and down the ulnar side of the left arm to the fingers. This pain he describes as being generally characterised by a sense of constriction, as though the heart were being compressed in a powerful grasp, but occasionally it is stabbing. It is always to be induced by exertion, and thus effectually prevents muscular effort, but at times it comes on at night, waking him from sleep, when the paroxysms are especially severe. When an attack occurs as the result of exertion he is compelled to stand still, and all movement is impossible until it has passed off, which it does after a period of rest. Accompanying the seizure is a fear of impending death, which, whilst prompting him always to carry about a paper on which is written his name and address for purposes of identification, is not, so far as I can gather from him, of the intensity which is sometimes described; at the same time, since he volunteers the statement about the paper, it must be very real. I have not seen him in one of these attacks, and therefore am not able to describe them to you as they appear to an observer, but they seem to be followed by a good deal of prostration. There is no especial complaint of dyspnœa at the time of the attack, though there is some shortness of breath otherwise.

Physical examination reveals the ordinary signs of aortic disease without mitral complication.

The history thus briefly related recalls in its essentials the general features of a case of angina pectoris as commonly described, and it is to its chief points that I wish to direct your attention.

You will first notice that the man looks ill. This is a point of importance. Angina is a disease which of all others inspires alarm and anxiety, and their traces are clearly shown upon the face of its victim.

The seat of the pain is behind the lower end of the sternum; this is the usual position, but by no means always so. It may be at the ensiform cartilage, the upper part of the sternum, or over any part of the præcordium; or again in rare cases it may be localised in those parts to which it more commonly radiates. A form of angina also occurs in which pain is entirely absent—angina sine dolore—and these cases, it may be remarked, are amongst the most serious, as I shall have occasion to refer to later on. It is therefore needful to bear in mind that pain, whilst often the prominent symptom, and placed in the foreground by the patient, is not a necessary concomitant of this disease, and its absence is not to be regarded as contra-indicative of angina.

The character of the pain is worth a moment's attention. This man tells us that it is constrictive, or resembles a weight oppressing the chest; this constrictive nature of the pain is one of its especial features and one upon which it is proper to lay stress; but, as you will have learnt from the history, it is not always so, but may be stabbing. Of its terrible nature you are sufficiently aware, and I need not labour it further. Professor Osler remarks that the resources of language have been taxed to describe it; such is the impression which it makes upon the sufferer.

A remarkable feature is the radiation. Commonly it is as described by this patient, to the left shoulder and along the ulnar aspect of the left arm, following the distribution of the ulnar nerve; but cases are recorded in which the radiation has been to the right arm, back of the neck, or even to the leg. In rare cases it may pass from the periphery, where it has begun, to the pericordium, and thus reverse the usual order. But, again, radiation, though common, is not essential, and may be absent.

In the case which I have just read to you the attacks are accompanied by a fear of the occurrence of death, and you will have noticed that the form in which the possibility of death presents itself to the patient is that it would be sudden, and before warning could be given, otherwise there would be no reason to carry about his name and address in a place like London, where solitude is not an every-day experience. This sensation of impending death is another of the special features of the anginal paroxysm, but it is not always present, nor is it necessarily accompanied by pain. It is the mental side of the attack, and seems to be connected with centripetal stimuli arising in an organ associated in the mind of the patient with one of the prime sources of life, and is obviously not a mere result of a painful sensation.

"During the paroxysm he is compelled to stand still"; this attitude of immobility is characteristic, the patient during an attack preserves the same posture which he had assumed at the onset; this is generally upright; lying down is said to intensify the suffering, a point which it is well to bear in mind in the question of treatment. Occasionally a patient finds ease in movement, therefore it is not to be inferred that because the attacks are not accompanied by immobility they are necessarily destitute of an organic basis.

There is one feature in the case which I have read to you upon which I wish to lay especial emphasis: a paroxysm, he tells us, can always be induced by an effort. This is a point of the greatest importance, and one which is distinctive of true coronary angina. I do not mean that exertion is the only way in which the symptoms can be provoked, but where they are definitely so produced true angina is to be feared.

In this case we have heard that paroxysms occur at night and may wake him from his sleep, but the diagnosis is not affected thereby; a paroxysm of true coronary angina can always be induced by a muscular effort, though not of necessity by all forms of effort, and where it is not so caused some doubt as to the diagnosis is permissible. As to the reason of this I shall have something to say by and by, but it may be here remarked that the great cause of the anginal seizure is an increase in the demand upon the heart for work, and anything which causes this may bring on an attack.

Among these causes are two chief and many others subsidiary; the most important are muscular exertion and mental emotion; but troubles of digestion appear to play a prominent part in some cases. It will be easily understood that a certain train of symptoms directly due to increased cardiac work may be produced in a multiplicity of ways; the one common factor underlying them being an excess of work demanded of the heart itself acting at a disadvantage.

The main ætiological factor in angina is arterio-sclerosis, and others such as heredity, gout, alcohol, and syphilis are probably only of importance in so far as they predispose to disease of the vessels; but this is not all, for arterio-sclerosis is common enough while angina is rare, and among the class of hospital patients still more infrequent. It is evident, therefore, that there is something, at present unknown, which acts either along with the vascular degeneration or directs it into a special channel.

As might be expected, the disease is more common among males than among females, and at the age when arterial degeneration is most frequently met with, that is, during the second half of middle life, but a case as early as twelve is on record.

Descriptions of the phenomena met with in an attack of angina vary considerably in their details, and this is not remarkable when it is remembered that the disease is rare and it is even more unusual to be present during a paroxysm; moreover, the attendant circumstances are such that close observation may be difficult or impossible—attention being more centred upon relief of symptoms than upon nice examination of physical signs.

The pulse varies in character in different cases; it may be accelerated or slowed, or it may be altogether unaltered. Sometimes it is weak and irregular. The general tendency is towards the latter condition, and it seems probable that much depends upon the violence and duration of the paroxysm. Where these are great the pulse and, of course, the heart, show signs of weakness or exhaustion. We are still without definite observations as to the blood-pressure during a seizure; the general opinion points to a rise in blood-pressure being the rule, but to this there are certainly exceptions, and writers upon the subject have recorded cases in which it has undergone no alteration. Examination of the heart, again, has produced uncertain results. Its action and sounds may be unaltered, but often evidence of weakness develops as shown by a gallop rhythm or embryocardia.

In a typical anginal attack respiratory features are absent; there may be a sensation of choking, but there is no dyspnœa. On the other hand the patient may fear to breathe and periods of apnœa result. This must not, however, be regarded as invariable, as mixed cases are frequently met with. The important point is that dyspnœa is not a necessary part of a true anginal paroxysm, and where breathlessness is prominent it indicates myocardial or renal changes.

Flatulence and vomiting occasionally occur, and it is of interest to note that in such cases, with pain located at the epigastric notch, confusion may arise, and a diagnosis of dyspepsia be made.

Vaso-motor effects, such as coldness and pallor of the extremities, are usual, but are less marked than in the hysterical form. Loss of consciousness is rare, but syncope is met with at times.

Such, then, are the symptoms of angina pectoris, from which it will be gathered that the clinical picture is one of great variability in detail; scarcely one feature can be said to be constant, but amongst them perhaps the tendency for an attack to be precipitated by muscular effort remains the most reliable. I do not propose to discuss points of diagnosis. In any case of supposed angina everything must be taken into consideration before a conclusion is arrived at, and it must not be too hastily assumed that all heart pains are necessarily anginal even if severe, for it may be taken as certain that the great majority are not, but have quite another origin, which must be carefully looked for and excluded. I refer to pains in connection with the stomach and projected to the præcordial region.

Prognosis is uncertain, and an opinion in this respect should always be guarded. Some cases seem to entirely recover and never have a return of the malady, whilst others are subject to recurrences only at long intervals, in addition to which the supervention of mitral complications may be followed by a cessation of the anginal paroxysms. Making allowance, however, for all this, it still remains unfortunately true that coronary angina is generally fatal. The subject of it is always on the edge of a precipice, over which he may be precipitated at any moment either suddenly or by a comparatively slow process of cardiac asystole. Any long series of cases can be divided, according to Professor Osler, into four groups:—

1. Sudden death without other manifestations of angina: These cases are not infrequent, and death is dramatic in its suddenness. Huchard relates a case of a financier who left the carriage in which he was riding to buy a newspaper at a stall. On glancing through it his eye fell upon a notice in which was recorded a depreciation in some stock in which he was largely interested. He was observed to become extremely pale, but got into his carriage, sat down beside his wife, and died with his eyes fixed upon the paragraph without making a movement or uttering a sound.

In this form death, when it occurs, is instantaneous, and may be quite without warning, as Huchard says the patient dies standing upright. It is highly probable that many of the instances of sudden death commonly regarded as the direct result of aortic disease are really due to the form of angina included under this group.

2. Death in the first well-marked paroxysm: Here death occurs during the first attack in a man apparently in good health. A classical instance of this kind is that of the celebrated Thomas Arnold, of Rugby.

3. Recurring attacks over months or years: This class includes the great majority of cases of angina. The attacks are repeated at variable intervals. Muscular exertions or emotions of any kind may bring about a seizure, and, as in the case of John Hunter, the patient goes about with the full knowledge that he carries his life in his hand, or what is more distressing, knows that it may be at the mercy of others.

4. Rapidly repeated attacks over a period of days or weeks, with the development of a state of cardiac asystole: Here the patient is seized with a paroxysm whilst in apparently good health; the attack passes off, but is repeated at more or less frequent intervals. Cardiac weakness supervenes, and the sufferer dies in a condition of extreme distress.

From considerations which I have put before you it is evident that the term angina is one which admits of much elasticity in its use, and therefore it will be well to have clearly in mind what one means by the expression before discussing its pathology.

I have hitherto spoken of angina pectoris as though it were a disease in itself, that, whilst very convenient, is not strictly accurate; it is a symptom and not a disease; and here confusion is possible, for as symptoms may be entirely absent and sudden death occur without warning in a man otherwise healthy, it is clear that they are really a subordinate part of the whole, since they may or may not be present, and that the idea which underlies the term angina is its fatality, and the possibility or probability of the occurrence of death more or less sudden. If a pain in the region of the heart is called angina, the idea conveyed by it should be that it has the grave significance referred to; if not, the expression has no more meaning than " cardialgia " or " neuralgia." A man complains of agonising pain about the heart; the question to be decided is what is the nature and effect of the lesion underlying it, and what is its bearing upon that man's chances of life or death. This is the point of view to which I wish to direct your attention pathologically; the patient will not die of the pain or angor, and from the four types of case which I have just quoted to you the inference is to be drawn that the gravity of the disease is not necessarily in proportion to the number of attacks which occur, but within limits rather the reverse, for recurrent attacks signify that the lesion which underlies the more obvious symptoms is still short of the full extent of its possibilities. After inquiring what is the pathological lesion which underlies the major phenomenon, and how it brings this about, we can then see if it is sufficient also to account for subordinate symptoms.

I do not propose to discuss the numerous theories, of which eighty are on record, which have been put forward from time to time to account for the phenomena met with in angina pectoris, nor is a clinical lecture of this kind a fitting place to do so, but to put before you the one which seems to best explain the facts.

Amongst the many theories, one pathological fact seems to be generally accepted; in most cases dead of angina in which a post-mortem examination has been made, atheroma of the coronary arteries has been found, and where exception has been noted there is some ground for the belief that it may have been overlooked, and that more exhaustive search would have revealed its presence; at the same time, it is conceivable, as I shall have occasion to point out later on, that some of the symptoms of coronary lesion may be reproduced in another way. But whilst accepting coronary sclerosis as the pathological basis, there still remains much that is obscure and requires further elucidation.

A series of post-mortem examinations at once presents a difficulty; coronary sclerosis and its resulting changes in the heart-muscle are common enough, but angina is infrequent; moreover the lesion is of everyday occurrence in hospital patients, in whom, as I have already said, angina is particularly rare. It is very difficult to reconcile this apparent contradiction, and before going further I must remind you of certain features of the coronary circulation and of the heart's action.

I need hardly say that the main nutritive supply of the heart is carried by the right and left coronary arteries, and upon the patency of these ultimately depends the maintenance of the cardiac action; at the same time it has been shown experimentally that the heart-muscle can be effectually nourished by means of the Thebesian orifices in its walls, the blood returning by way of the coronaries. Between the two main vessels there is a certain amount of anastomosis, by which the circulation can be carried on in the event of occlusion of one of them, provided that the process is sufficiently gradual.

The results of occlusion of the coronary arteries vary with the size of the vessel affected, and with the rate at which the process takes place. Sudden blocking of a main vessel or of one of the larger branches is followed by sudden death, the organ going into fibrillary contractions; but where a small branch is so occluded, infarction results, of which the patient may recover, a small fibrous scar being left. Gradual reduction of the lumen, however, is followed by fatty and sclerotic changes in the myocardium with atrophy of the muscle fibres, which shows itself during life by progressive cardiac weakness; thus the results of occlusion vary both pathologically and clinically with the manner in which it is brought about, and also with the size of the vessel occluded.

A very suggestive feature is that the heart is an automatic organ generating and distributing its own stimuli by means of more or less specialised portions of its tissue, and thus it is probable that a very close relationship between function and blood-supply obtains, in so far as two functions, stimulus production and contractility, have to be subserved, and a certain minimal supply is needful.

When the body is at rest the heart liberates only a part of the energy of which it is capable; the remainder is called the reserve force, and is available to meet requirements which are liable to constant variation according to the needs of the body. This reserve power, which must ultimately be dependent upon an adequate blood-supply, is called upon whenever a demand is made upon the heart for work in excess of what is required of it when the body is at rest, and must be accompanied by an increased blood-flow to the cardiac muscle in proportion—broadly speaking—to the work demanded of it, and if this fails it is reasonable to suppose that symptoms will arise which will depend for the precise character which they will assume upon the manner in which the deficiency in blood-flow has been brought about. Now the difference between the amount of energy liberated during maximum activity and that which is sufficient when the body is at rest is very great—no less than thirteen times the minimum amount—and in order to provide for this amount of energy there must be a very considerable working margin of blood-flow available between that which is needful during periods of comparative rest and that during full activity; that is to say, the volume of blood delivered to the heart varies with the necessities of the moment, and it is probable that it may undergo considerable fluctuations so long as an activity in excess of the available blood-supply is not required.

One point more I must recall to your minds; the proper nutrition of a muscle presupposes, amongst other things, not only an amount of blood adequate to bring material for the supply of energy, but also a flow sufficient in rate and volume to carry off the waste products of metabolism, and failure in these respects may be followed by results serious in proportion to the organ affected.

It will thus be apparent that the heart is more than a mere force-pump to which it is often compared, but is a delicately balanced piece of mechanism whose automatically regulated activities depend upon a blood supply adequate to support an enormous energy production, and which, subject to the control of the nervous system, maintains a nice equilibrium between demand and supply by means of its reserve power, whilst itself is also subject to the same conditions. This does not mean that reserve power is only a question of blood flow; in the long run the amount of available reserve is modified by the nature of the calls to which the organ has been accustomed; but that in order to perform a certain amount of work a minimum blood flow is needful, and if this fails symptoms will arise.

What now is the bearing of these considerations upon the subject before us? Much light is thrown upon the matter by a remarkable phenomenon to which the name intermittent claudication has been applied. Intermittent claudication is described by Professor Osler as follows: There is "an affection in the horse in which, after being driven for fifteen or twenty minutes, the animal stops, the hind legs get stiff, and soon it is unable to stir. It may fall down and apparently be in great suffering. In from half an hour to an hour it will recover and will go on comfortably for another fifteen minutes, and then an attack recurs. In such cases, post-mortem, the artery of the affected limb has been found blocked with a clot, or, when both hind legs have been involved, the abdominal aorta has contained a thrombus." A similar train of symptoms has been described in man accompanying obstruction to the main arterial supply to a limb, numbness, tingling, paresis, or transient paralysis are the usual symptoms, and arteriosclerosis is generally present. In both these cases the phenomena observed evidently owe their existence to arterial obstruction, by which a relative ischæmia is produced; a condition in which the demand for arterial blood is in excess of the possibilities of supply, and it is worthy of note that the obstruction is situated in the main arterial channel so that the deficiency in the blood supply is a deficiency in bulk and not in detail.

If you will now transfer this idea of claudication from the extremities to the heart, a very probable explanation of the phenomena of angina pectoris will be apparent, a block exists in the main blood supply to the ventricular walls, commonly the left, of such a nature that the demand for a greater nutritive supply, arising from some cause which induces an increased activity of the organ, cannot be met, and symptoms appear comparable to those occurring when a similar state of things exists in the limbs. It should be noted that the most severe cases of angina are met with when there is sclerosis at the root of the aorta, that is, where the block is at the orifice of the coronary artery, and so reproduces the condition of affairs in intermittent claudication. Moreover the lesion must be one which reduces the lumen of the vessel; it is not sufficient that the wall is simply atheromatous and rigid. At the same time angina and cardiac insufficiency are not precisely the same thing; insufficiency follows destruction of the muscle fibres in detail; angina, where there is a reduction of the blood supply *en masse*, and thus the conditions needful to bring about cardio-sclerosis and those needful to cause angina are somewhat different, but as a similar process is at work in both, and it is only a question of position, mixed cases are to be expected. Here then is a reasonable explanation of the phenomenon of sudden death in angina; the heart as regards its blood supply is in a state of unstable equilibrium; under a stress a relative ischæmia is produced in which the blood flow falls below the minimum demanded by the organ for efficient contraction under the conditions in which it finds itself, a large part or even the whole of the left ventricle is affected, and paralysis of the organ and sudden death ensue.

But, seeing the lesion must be a progressive one, how does it come about that sudden death may occur without warning. Recurrent symptoms following a progressive lesion would have been expected. I must remind you that this is precisely what usually happens, and where it does not there are two ways of accounting for the exceptions. One is that the primary lesion is unusually slow in forming, and so gives time for the development of a collateral circulation, failure occurring when the resources of this are at an end. In the case of Thomas Arnold "there was but one coronary artery, and that, considering the size of the heart, was of small dimensions. It presented also a slight atheromatous deposit an inch from its orifice." Here an anastomotic channel was essential to life, and "a slight atheromatous deposit" in the main supply finally upsets the nicely adjusted balance. In such cases it is not a great assumption to make that the anatomical conditions necessary for the opening up of anastomosis between the two coronaries vary in different cases.

A second explanation may be also offered. I have referred to the margin of blood supply between maximum and minimum activity; where two lesions take place together such as obstruction to the main channel and occlusion of its ultimate branches, each may be expected to produce its own effects, but the result may be that the balance normally obtaining between the amount of blood flow and the total mass of muscle to be supplied may not be seriously disturbed until the one, advancing relatively faster than the other, finally produces a state of things similar in effect to that just mentioned, together with a like result; or, when the balance is not so well maintained, a mixed case ensues.

Pain in Angina Cases.

What now is the relationship between the attacks of anginal pain and coronary occlusion? If I have made myself clear so far, you will have reached the conclusion that the cardinal factor in the syndrome is obstruction to the cardiac blood supply, and though much yet remains uncertain and a matter of conjecture, it yet seems probable that the lesion responsible for one symptom is also at the root of them all; that is to say, the pain is in some way bound up with the defective circulation, and has much the same nature and origin as that found in intermittent claudication. It is, as I have said, a subordinate symptom notwithstanding its severity and the distress and disability to which it gives rise; the patient does not die of it, but of the coronary disease; in other words, the pain is a warning that a particular form of defect in the cardiac circulation exists; that it recurs from time to time may be regarded as showing that that defect has not yet reached an extreme phase, and in so far as some cases appear to recover and have no return, it may be supposed either that the lesion has become absorbed or that the anatomical conditions prevailing have allowed anastomotic channels to compensate for a lesion which has become stationary, ending in the establishment of a more or less normal relationship between blood flow and muscle bulk.

Precisely why the pain has its peculiar constrictive character is uncertain; it has been suggested that it is the result of contraction of the intercostal muscles, a motor reflex, akin to the rigidity obtaining over the site of a peritoneal inflammation. It seems to me more likely that the pain is an expression of stimuli produced as the result of irritation of the nervous structures of the heart consequent upon the imperfect removal of products of metabolism by an insufficient blood-stream.

The best explanation of the radiation that has been put forward appears to be that it is due to stimuli, arising in the heart, being conducted to that segment of the cord which gives origin to the ulnar nerve, to the distribution of which it is referred in the same way as is familiar in other parts of the body. Some variation no doubt exists in the resistance offered in the cord to the spread of the stimulus, and depending upon the nature of this will be the exact distribution of the radiated pain.

I have only time to refer very briefly to a possibility which I have already hinted at. There is a form of angina in which its manifestations appear to be bound up with marked general vaso-motor phenomena, where it is possible or even probable that the cardiac symptoms are the outcome of an extension of vaso-constriction from the peripheral to the cardiac vessels, and it may be that in some cases coronary atheroma is associated with spasm; but I cannot further pursue this interesting side of the subject.

